# Predictors for time to recovery from sever acute malnutrition among under-five children admitted to therapeutic feeding unit at Dubti referral hospital, Afar region, Ethiopia

**DOI:** 10.1186/s12887-021-03043-x

**Published:** 2021-12-10

**Authors:** Awoke Seyoum Tegegne, Denekew Bitew Belay

**Affiliations:** Department of Statistics, Bahir Dar University, Bahir Dar, Ethiopia

**Keywords:** SAM, Average recovery time, Acute malnutrition, Global malnutrition, Recovery rate

## Abstract

**Background:**

Currently, about 165 million children are categorized under malnutrition and 51.5 million suffering from acute malnutrition in world wide. Hence, the objective of current study was to assess the recovery time and its predictors of children under five from severe acute malnutrition admitted to Therapeutic Feeding Unit at Dubti Referral Hospital, Afar region, Eastern Ethiopia.

**Methods:**

Institutional based retrospective cohort study was conducted on 650 inpatient children with SAM admitted for therapeutic feeding unit whose treatment was from March to April/2017.

**Results:**

The result in current investigation indicates that the average recovery time from SAM was found to be 21 days (95% CI; 21.23–25.77), *p*-value = 0.035). A Cox proportional hazard regression model revealed that Weight of a child at birth, gestational age of a child, working status of a child at admission birth order of a child, mother’s BMI, mother’s level of education, mother’s stature, mother’s occupation, mother’s age, mother’s marital status, mother’s nutritional status, house hold income in ETB, family size in HH, number of under-five children, the type of toilet used in HH, source of improved drinking water, type of cooking fuel, ownership of livestock, age and weight of a child at admission had statistically significant association with the variation of average recovery time of children from SAM.

**Conclusion:**

Male children under severe acute malnutrition, rural children, children with different additional diseases and children who did not get mothers’ breast milk at least in the first six months after birth and children who did not get vaccination are groups at risk and needs intervention and special attention to be recovered with short period of time. Children from low income family, who did not get improved drinking water, without moderate cooking fuel and a child from larger families were groups at risk in recovery time from SAM.

## Background

Currently, an estimated number of 165 million children are categorized under malnutrition (stunting, wasting and underweight) and 51.5 million suffering from acute malnutrition in world wide. The hazard leads to morbidity and mortality of childhood because of malnutrition and this further leads to worse in children intellectual growth, adult efficiency and may rise in the hazard of enlargement for a firm of disease in parenthood [1]. Acute malnutrition attributes to 875,000 deaths of under five children and this accounts for 12.6% of all deaths in under five children [2].

Enlightening of diet is a worldwide primacy, with the aim of reducing global malnutrition by 2030 that had been incorporated in the 2015 United Nations Sustainable Development Goals [2, 3]. Malnutrition in children remains main community well-being risk in several low income nations especially, Sub-Saharan Africa and it remains to be the supreme essential determinant factor for physical and mental growth obstacle, progression of disease and finally large mass of deaths in young children [4]. Malnutrition consists of Severe, Chronic or Sever Acute Malnutrition (SAM).

Malnutrition is said to be Sever Acute Malnutrition(SAM), if body mass index is less than 3 standard deviation or weight for height ratio is less than 70% [5]. Among children suffering from SAM, the widespread are found in Southern and Southeast Asia and Sub-Saharan Africa [6]. In countries like India, Indonesia, Kenya and Ethiopia, SAM not only exist in sudden situations, but also it is existed in stable settings [7]. In these countries, SAM is perceived as a condition of community-spirited disasters rather than progressive and health importance [8]. The risk of SAM further leads to long-term economic and social burden in such countries [9].

The reason for this, may be owed to limitation of the wellbeing conditions and occurrence of bottle neck obstacles at lower levels of healthcare surroundings [10]. This might be due to limitation of health care settings and occurrence of different challenges at local levels of low resource healthcare surroundings [11].

Ethiopia is one of the Sub-Saharans Africa in which many children are suffering from SAM and all activities with treatment outcomes of SAM in the healthcare surroundings could be assessed. Few studies had been conducted in Ethiopia about SAM and as far as the investigators knowledge is concerned, there is a scarce of studies about predictors of children’s survival time from SAM, admitted to therapeutic feeding unit in the study area.

Because of its cost effectiveness, an integrated communal based control of SAM is currently well-thought-out as standard for care of basic SAM [12]. A research investigated on control of clinical and public trials in Ethiopia approved that community based management program should be conducted in large-scale implementation by government’s health institutions [11].

Previous research conducted on outpatient therapeutic feeding unit program in southern Ethiopia, revealed that there is a great challenge in conducting an integrated communal based program for SAM, especially remotest areas in the country [13]. The previous investigation also revealed that, only 39% of an Integrated Community based Outpatient Therapeutic program (ICOTP) fulfill the local and World Health Organization (WHO) guideline. The previous study also revealed that, recovery of children from SAM based on WHO guidelines using outpatient therapeutic feeding unit is not an easy task to be practically implemented [10, 13].

Another study highlighted about the challenges of scaling up of ICOTP in to the lowest levels of the health institutions and needs for an investigation of the recovery time of children enrolled for SAM based on the national and WHO recovery guideline [14]. Another investigation conducted in Ethiopia, stated that, household level treatment for SAM is no longer effective and for the program to be successful. On the other hand, access to potentially life-saving care for every child enrolled at health facilities using an integrated Mid-Upper Arm-Circumference (MUAC) in primary health care surroundings is recommended to be conducted [15]. Most of the previous researches focused on the experience and challenges of applying WHO’s guideline and did not investigate socio-demographic and economic factors affecting recovery time of children from SAM.

The objective of current investigation was therefore, to assess recovery time from SAM and its determinant factors amongst children under five admitted to therapeutic feeding unit at Dubti Referral Hospital, Afar region, Eastern part of Ethiopia. The treatment site in the country is known by its shortage of food for community.

## Methods

### Setting and study design

The study was conducted using institutional based retrospective cohort data recorded from March to April, 2017 at Dubti Referral Hospital in Afar region, Eastern Ethiopia. The hospital provides inpatient services for primary health care for children with SAM in the region. The study employed secondary data collected by health staffs.

#### Source of data

The data from the chart of each child was collected by the health staff to assess the visibility of primary health care surroundings. Hence, current study used secondary data collected from health institution (Dupti Referal Hospital).

#### Data collection procedures

During data collection, health staffs and clinical nurses were participated in collecting secondary data from cards of patients and a public health expert was assign as a supervisor and orientation was given for data collectors about the variables under current investigation. The data collection format was developed by the investigators in consultation with the health staff, considering the national and WHO protocol for the management of SAM.

### Eligibility criteria

All children under five with SAM that have been admitted as inpatient for primary health care and treated at therapeutic feeding unit (TFU) in Dupti referral hospital from March to April, 2017 were eligible for the study. The study included children who got treatment for SAM, children admitted for MUAC value of B110 mm or bilateral pitting nutritional edema, children who tested their appetite and passed the test and children without treatment difficulties in the hospital were included in current investigation. Study participants were categorized as Marasmus (children with nutritional edema) and kwashiorkor (children without nutritional edema).

#### Sample size and sampling procedure

To compute an appropriate sample size, an Open Epi version 2.3 was used with the assumption that the proportion was recovered in both Marasmus and Kwashiorkor group children at 95% CI and 5% marginal error. Using this approach, the smallest size for both groups was 590. To compensate for potential losses of information for missingness, 10% of the sample was added. Hence, about 650 children who got treatment in the hospital were taken as a sample in both groups (Marasmus and Kwashiorkor).

From the selected hospital, eligible children with SAM were identified using patients’ card and list of individual in sample selection procedure was developed as sampling frame. Proportional allocation was employed for groups namely Marasmus and Kwashiorkor groups. Finally, individual participants were selected using systematic random sampling using patients’ card number.

#### Data cleaning and checking its quality

Both the data collectors and the supervisors got two days training/orientation about the variables under current investigation. There was a close follow ups or supervisions of the data collection by public health experts. After data collection, data were entered in to Epi info version 6 by data encoders and cleaned all the errors created during data entry. This was done by the investigators with one health staff (to check the appropriate use of medical terminologies under this investigation). Hence, Data were cleaned before data analysis and close follow ups were there in collecting secondary data. Pilate test was conducted to test the relevancy and quality of instruments used for data collection. The completeness and consistency of questions in this regard was also checked on 45 sample data and proper amendments were included after getting feedbacks from pilot test.

### Study variables

#### Response variable

The response variable for current investigation was average recovery time from Severe Acute Malnutrition (SAM). Malnutrition consists of stunting, wasting and under-weight.

#### Predictor variables

The predictor variables consists of breast feeding history (yes, no), weight of a child at birth, gestational age of a child (extremely preterm (less than 28 weeks), very preterm (between 28 and 32 weeks, late preterm (between 32 and 37 weeks), working status of a child(yes, no), birth order of a child (first, 2–4, > 4), parent’s level of education(no-education, primary, secondary and above), mother’s stature(normal, short), mother’s occupation (household wife, Gov’t employee, Private), mother’s age categories(15–24, 25–34, 35–44, > 45), mother’s marital status(married, others), house hold income in ETB(< 500, 500–1000, > 1000), age ranges between children(< 2 years, > = 2 years), family size in HH(< 5, 5–10, > 10), number of under-five children(< 2, > = 2), Source of improved drinking water(yes, no), type of cooking fuel(moderate, traditional), adequate access of HH food (yes, no), sex of a child (male, female), Residence area(rural, urban), malnutrition type (stunted, wasted, under-weight), age in months and weight in kg, type of house families live(moderate, traditional).

#### Measurements of time variant and in-variant covariates

MUAC was measured on the left upper arm of a child with the arm hanging down of the body and. The value obtained in this way was recorded to the nearest value of 1 mm. First, desire for food on outpatient children was tested weekly for children enrolled in the program. A child was said to pass the desire for food test, if she or he was able to eat the food ready to use therapeutic food (RUTF) recommended for her or his body weight. On the other hand, children who failed the appetite test conducted weekly were referred to inpatient care [16]. Depending on their weight, the national protocol for management of SAM directed children to receive different number of RUTF [17, 18]. The progression of MUAC in mm/day and weight increase in g/kg/day was computed for all inpatient children enrolled for SAM in the hospital.

The nutritional status of children was also assessed and categorized as malnutrition or not. Malnutrition consists of Severe, Chronic or Sever Acute Malnutrition (SAM). Malnutrition is said to be Sever Acute Malnutrition(SAM), if body mass index was less than 3 standard deviation or weight for height ratio is less than 70% [5].

For children with kwashiorkor, the change of MUAC and the change in weight were computed after the edema has been determined. Children admitted for treatment was categorized as new (admitted for the first time or after 2 months of recovery) or re-admitted (admitted within two months of recovery). A child was considered as discharged from treatment because of his/her recovery from SAM, move/transfer to other health facilities, defaulted from treatment or death, Body weight was measured using a digital weighting scale and for children whose age was less than three months. Here, a 25-kg hanging spring scale graduated by 0.1 kg was used.

Follow up characteristics of children with SAM related to lose of edema and weight gain in the first phase was conducted on this cohort on the 4th, 10th day and day 10 was considered as cut of point to pass to the second phase. A child was recovered from SAM if he/she obtained 15% of weight expected (target weight) and become free from edema [19].

#### Admission medication

In the admission procedure, amoxicillin was given for one week. At a time admission, vitamin A was given for all children. Vitamin A was also given for children at the fourth visit, if they become free from edema and for those children who did not take it for the last 6 months after birth [20], Measles vaccine was given on the fourth visit, and Deworming was given on the second visit [19].

### Data processing and analysis

Data were entered in to Epi info version 6 statistical software and then exported to SPSS version 23 for analysis. Data was described using percentages for qualitative data and averages for continuous variables. To test the association between categorical variables, Pearson chi-square test was used.

### Statistical models used for current investigation

Kaplan Meier estimator which is a non-parametric survival curve was applied in this invesitigation [20]. If n subjects are on test and ordered the observed lifetimes for these n individuals from t_(1)_ to t_(n)_ and r individaules are cured, then ordered cure times are t(1),…, t(r), where r ≤n. The probability that an individual cures during the small time interval is estimated by $\frac{{\mathrm{c}}_{\mathrm{j}}}{{\mathrm{n}}_{\mathrm{j}}},$ where c_j_ is censored time. The chanace of surviving through the interval from t_(k)_ to t_(k + 1)_, and all preceding intervals lead to the Kaplan-Meier estimate of the survival function, which is given by:

1 $${\hat{\mathrm{S}}}_{\left(\mathrm{t}\right)}=\prod_{\mathrm{j}=1}^{\mathrm{k}}\frac{{\mathrm{n}}_{\mathrm{j}}-{\mathrm{c}}_{\mathrm{j}}}{{\mathrm{n}}_{\mathrm{j}}}$$

The log rank test was used to compare two or more independent survival curves and usefull for non-overlapping survival curves. The log rank test statistic for comparing two groups is given by:

2 $${\mathrm{X}}_{\mathrm{LR}}=\frac{{\left(\sum_{\mathrm{i}=1}^{\mathrm{m}}{\mathrm{c}}_{1\mathrm{i}}-\sum_{\mathrm{i}=1}^{\mathrm{m}}{\hat{\mathrm{e}}}_{1\mathrm{i}}\right)}^2}{\sum_{\mathrm{i}=1}^{\mathrm{m}}\hat{\mathrm{v}}\left({\hat{\mathrm{e}}}_{1\mathrm{i}}\right)}$$

where m is the number of rank ordered cures times, c_ji_ is the number of people experiencing the event at time t_(i)_ in group j, n_ji_ is the number of people at risk in group j at time t_(i)_, c_i_ is the total number experiencing the event in both groups, ${\hat{\mathrm{e}}}_{\mathrm{ji}}=\frac{{\mathrm{c}}_{\mathrm{i}}{\mathrm{n}}_{\mathrm{ji}}}{{\mathrm{n}}_{\mathrm{i}}}$ is the estimated expected number of individuals experiencing the event at t_(i)_ in group j, ${\hat{\mathrm{v}}}_{\left({\hat{\mathrm{e}}}_{\mathrm{ji}}\right)}=\frac{{\mathrm{n}}_{1\mathrm{i}}{\mathrm{n}}_{2\mathrm{i}}{\mathrm{c}}_{\mathrm{i}}\left({\mathrm{n}}_{\mathrm{i}}-{\mathrm{c}}_{\mathrm{i}}\right)}{{{\mathrm{n}}_{\mathrm{i}}}^2\left({\mathrm{n}}_{\mathrm{i}}-1\right)}$ is the estimated variance of ${\hat{\mathrm{e}}}_{\mathrm{ji}},$ n_i_ is the number of individuals at risk in both groups 1 and 2 just prior to event time t_(i)_.

A Cox-proportional hazard model was also used for exploring the relationship between the survival time and several explanatory variables. The hazard function is proportional to the instantaneous risk at any time t, given that an individual has lived at least t_0_ up to time t and indicated by h(t) and is defined as follows [20]:

3 $${\displaystyle \begin{array}{c}\underset{\kern2.75em \Delta \mathrm{t}\longrightarrow 0}{\mathrm{h}\left(\mathrm{t}\right)=\lim}\kern0.5em \frac{\mathrm{P}\left[\mathrm{t}\le \mathrm{T}\le \mathrm{t}+\Delta \mathrm{t}|\ \mathrm{T}\ge \mathrm{t}\right]}{\Delta \mathrm{t}}\\ {}\mathrm{h}\left(\mathrm{t}\right)=\mathrm{P}\left(\mathrm{t}<\mathrm{T}<\left(\mathrm{t}+\Delta \mathrm{t}\right)\ |\ \mathrm{T}>\mathrm{t}\right)\\ {}=\frac{\mathrm{f}\ \left(\mathrm{t}\right)}{1-\mathrm{F}\left(\mathrm{t}\right)}=\frac{\mathrm{f}\ \left(\mathrm{t}\right)}{\mathrm{s}\left(\mathrm{t}\right)}\end{array}}$$

Since h(t) is also equal to the negative of the derivative of   ln(S(t)), we have the useful identity:

4 $$\mathrm{S}\left(\mathrm{t}\right)={\mathrm{e}}^{-{\int}_0^{\mathrm{t}}\mathrm{h}\left(\mathrm{t}\right)\mathrm{dt}}$$

If we let H (t) = ${\int}_0^{\mathrm{t}}\mathrm{h}\left(\mathrm{t}\right)\mathrm{dt}$ be the cdf (cumulative hazard function), we have  S(t) = e^−H(t)^.

Then the general hazard regression model is:

5 $$\mathrm{h}\left(\mathrm{t},\mathrm{x}\right)={\mathrm{h}}_0\left(\mathrm{t}\right){\exp}^{\left(\mathrm{x}\upbeta \right)}$$

## Results

Out of the total 650 children in the cohort; 384 (59.1%) were males, most of study subjects 64.6% were rural residents. From all cohorts; 71.5% were newly admitted, 64.5% were edematous and 18.5% were wasted and the rest were both edematous and wasted. Among all children under malnutrition, 30% didn’t get their mothers’ breast milk, the majority of them (64.6%) were from rural area, 50.8% of them were at functional status, about 50.4% of the children were from the birth order of 2–4, 70% of them had additional disease history. As, it is indicated in Tables 1, 53% of the children were very preterm and 85.4% were born from short mothers, 68.8% of the children came from families in which their mother and father are not live together and 54.2% of them came from families with additional children with malnutrition (Ref. Table 1).

**Table 1:** Socio-demographic and baseline characteristics of study variables

Variables	Category	Frequency	Percentage
Sex of a child	Male	384	59.1
	Female	266	40.9
Weight of a child at birth	Small	350	53.8
	Average	220	33.8
	>average	50	12.4
Gestational age of a child	extremely preterm(less than 28 weeks)	96	14.8
	Very preterm(between 28 and 32 weeks)	345	53.08
	Late preterm (between 32 and 37 weeks)	209	32.2
Residence area	Rural	420	64.6
	Urban	230	35.4
Working Status of a child	yes	330	50.8
	no	320	49.2
Birth order of a child	first	165	25.4
	2–4	345	50.1
	> 4	140	21.5
Admission status	New	465	71.5
	Readmission	185	28.5
Malnutrition category of a child	Stunted (height to age)	425	65.4
	Wasted (weight to age)	120	18.5
	Under-weight (height to weight)	105	16.1
Breast feeding history	yes	455	70.0
	no	195	30.0
Additional disease	yes	455	70.0
	no	195	30.0
Parent’s level of education	No education	145	22.3
	Primary	305	46.9
	Secondary and above	200	30.8
Mother’s stature	Normal	95	14.6
	Short	555	85.4
Mother’s age	15–24	153	23.5
	25–34	150	23.1
	35–44	178	27.4
	> = 45	169	26
Mother’s occupation	Household wife	153	23.5
	Gov’t employee	328	50.5
	Private	169	26
Mother’s marital status	Married	203	31.2
	others	447	68.8
HH monthly income(ETB)	< 500	153	23.5
	500–1000	228	35.1
	> 1000	269	41.4
Age ranges between children	< 2 years	197	69.7
	= > 2 years	453	30.3
Family size in HH	< 5	153	23.5
	6–10	228	35.1
	> 10	269	41.4
No of under-five children in HH	1	133	20.5
	2	248	38.2
	> = 3	269	41.4
Type of toilet HH used	Improved	352	54.2
	traditional	298	45.8
Source of improved drinking water	yes	402	69.5
	no	198	30.5
Type of cooking fuel	Moderate	340	52.3
	traditional	310	47.7
Whether there is any other malnutrition in the HH	yes	352	54.2
	no	298	45.8
Appetite test	Pass	54	8.3
	fail	458	70.5
	Not recorded	138	21.2

In the treatment site, admission medication was also conducted for all children and 19.2% of children received Amoxicillin, 14.8% of the children received Ampicillin and the majority of the children (44.5%) received vitamin A.

Table 2 indicates that about half (50.8%) of the children with SAM didn’t start to reduce edema on the 4th day after getting treatment and 30.8% of the children with SAM had edema on the 10th day after getting treatment and 18.5% of children with SAM cannot pass to the second phase. Finally, about 38.5% of children with SAM didn’t gain > 5 g per day for three consecutive days.

**Table 2:** Treatment outcomes for under-five children admitted to Therapeutic Feeding Unit

Variables	Frequency	%
Fail to start in losing edema on 4th day	330	50.8
Presence of edema on the 10th day	200	30.8
Fail to pass to the second phase on the 10th day	120	18.5
Fail to gain weight > 5 g per day for three successive days	254	38.5

At the end of study period, the discharged status for children with SAM was assessed and out of the total 650 children in the cohort, 408 (62.89%) were recovered, 37 (5.7%) died, 205 (31.5%) defaulted, 9(1.4%) not recorded on their card and 57 (8.8%) transferred to other treatment sits. The nutritional recovery rate of this cohort was 3.56 per 100-person. The average nutritional recovery time was estimated to be 21 days (95% CI; (21.23–25.77).

In Table 3, it is indicated that the predictor variables and the variable of interest significantly associated each other with the value of chi-square and corresponding *p*-values. Hence, the chi-square and corresponding p-values were evidence for the predictors and response variables to be highly associated.

**Table 3:** Log rank test for categorical variables and average recovery time of children from SAM

Variables	Categories	n (%)	Average recovery time			Chi-square value	p-value
			Estimate	95% CI			
Sex	Male	384(59.1)	17.00	14.00	19.00	7.80	.035
	Female	266(40.9)	14.00	10.56	18.00		
Residence area	Rural	420(64.6)	27.00	23.00	31.54	7.78	.020
	Urban	230(35.4)	15.00	11.50	17.00		
Gestational age	Ex. preterm	265(40.8)	18.00	14.50	22.00	8.75	.032
	Very preterm	185(28.5)	15.00	11.00	20.00		
	Late preterm	200(30.8	10.15	6.54	13.50		
Diagnosis at admission	Edematous	425(65.4)	16.00	13.00	19.00	11.54	.043
	Wasted	120(18.5)	13.00	11.00	15.55		
	Both	105(16.2)	18.00	15.00	20.00		
Breast feeding history	yes	455 (70.0)	9.00	7.00	11.55	8.75	.033
	no	195(30.0)	17.00	14.00	19.56		
Vaccination history	yes	345(53.1)	10.54	8.45	12.55	7.58	.003
	no	305(46.9)	14.55	12.55	16.56		
Additional disease	yes	355(54.6)	16.00	13.00	18.56	9.75	.038
	no	295 (45.4)	8.50	6.00	11.00		
Working status of a child	yes	254(39.1)	15.54	11.00	17.00	8.89	.024
	no	396(60.9)	10.50	7.50	12.55		
Birth order of a child	first	300(46.2)	14.85	12.00	16.56	6.56	.045
	2–4	248(38.2)	8.56	5.85	10.55		
	> 4	102(15.7)	10.12	6.54	14.35		
Mother’s Nutritional status	Under nutrition	345(53.1)	21.50	18.56	24.55	9.55	.034
	Normal	195(30.0)	16.00	13.56	18.55		
	Obese	110(16.9)	18.02	13.42	24.51		
Parent’s education	No education	234(36.0)	15.05	12.00	21.61	8.54	0.037
	primary	253(38.9)	14.73	10.23	18.54		
	Secondary and above	163(25.1)	16.54	12.34	20.01		
Mothers Stature	Normal	95(14.6)	14.00	12.00	17.56	12.45	.035
	Short	555(85.4)	24.00	22.00	27.00		
Mother’s age in years	15–24	105(16.2)	12.00	9.55	22.55	12.50	.054
	25–34	245(37.7)	13.45	9.55	15.65		
	35–44	195(30.0)	14.24	8.92	15.06		
	> = 45	150(23.1)	15.93	11.16	18.82		
Mother’s occupation	HH wife	125(19.2)	18.05	15.00	20.55	9.55	.035
	Gov’t employee	200(30.8)	21.24	17.35	25.91		
	Private	325(50.0)	12.00	10.00	15.00		
Mother’s marital status	Married	203(31.2)	12.54	8.00	15.55	11.54	.003
	Others	447(68.8)	17.86	13.92	20.45		
HH monthly income(ETB)	< 500	125(19.2)	18.65	15.56	20.55	9.65	.004
	500–1000	96 (14.8)	14.56	12.00	16.56		
	> 1000	85(13.1)	11.45	8.45	14.56		
Age range between children	< 2 years	197(30.3)	15.21	18.98	25.76	8.43	0.072
	> = 2 years	453(69.7)	10.9	13.42	23.12		
Family size of HH	< 5	187(28.8)	11.45	9.83	15.34	12.21	0.021
	6–10	379(58.3)	14.02	12.34	21.45		
	> 10	84(12.9)	16.58	10.83	17.51		
No. of under five children	<=2	276(42.5)	10.24	8.45	13.40	9.45	0.045
	> 2	374(57.5)	14.25	10.23	18.41		
Use of improved Toilet by HH	yes	154(23.7)	12.06	11.23	18.45	10.43	0.025
	no	496(76.3)	15.28	10.34	17.53		
Source of adequate HH food	yes	308(47.4)	9.21	5.98	14.07	9.87	0.012
	no	342(52.6)	12.45	7.54	12.65		
Source of improved drinking water	yes	402(61.8)	9.21	5.98	14.07	10.2	0.031
	no	248(38.2)	12.45	7.54	12.65		
Use of moderate cooking fuel	yes	247(38.0)	12.54	8.48	15.04	9.57	0.032
	no	403(62.0)	15.98	11.65	18.34		
Type of house families live	Modern	430(66.2)	10.27	8.02	14.21	10.45	0.001
	Traditional	220(33.8)	13.89	10.45	15.25		
Appetite test	Pass	229(35.2)	12.45	9.45	15.45	12.23	0.032
	Fail	250(38.5)	13.62	10.12	16.51		
Average recovery time for all children			21	15.23	25.77		0.035

**Fig. 1:**
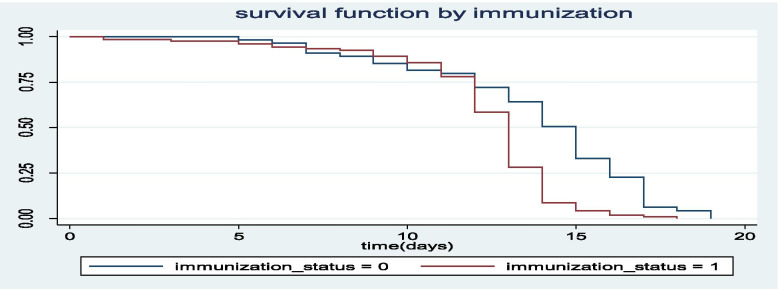
The Kaplan-Meier survival curves for under five children with SAM with immunization and without immunization

**Fig. 2:**
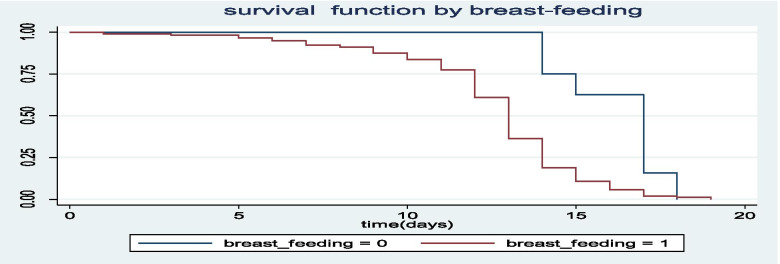
The Kaplan-Meier survival curves for under five children with SAM with and without mothers breat milk feeding

**Fig. 3:**
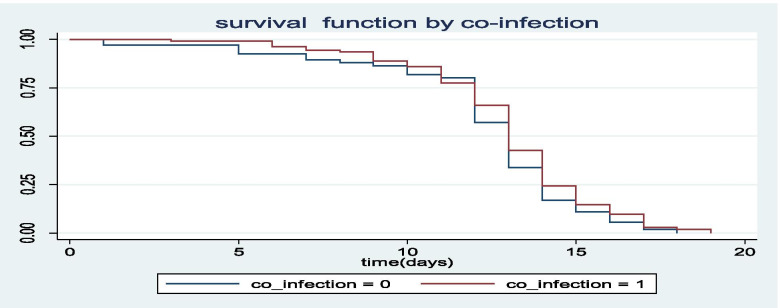
The Kaplan-Meier survival curves for under-five children with SAM with co-infected and free from co-infected with additional disease

The Kaplan-Meier survival curves for each study variable provide an initial insight for the shape of survival function. The Kaplan-Meier survival curves of three important covariates are indicated in Figs. 1, 2, 3.

Figure 1 indicates that those children who did not get immunization (immunization-status = 0) had longer average recovery time from SAM as compared to those children who got immunization (immunization-status = 1).

As it is indicated in Fig. 2, the average recovery time from SAM for children who did not get their mothers’ breast milk (breast –feeding = 0) was longer than those children who got mothers’ breast milk (breast-feeding =1).

Figure 3 indicates that the average recovery time from SAM for those children who were con-infected with additional disease such as HIV, Diarrhoea, Vomiting, Cough, Fever> 39’c, Anemia, Malaria, Pneumonia and TB (Co-infection = 1) was longer than those children free from such co-infected diseases (co-infection = 0). Hence, children with and without additional diseases had different average recovery time. The predictors of time to recovery from SAM in current investigation are indicated in Table 4.

**Table 4:** Predictors of time to recovery from SAM, Multivariate Cox Proportional hazard Model

Variables	Estimate	Hazard Rate(HR)		p-value
		Estimate	95% CI	
Intercept	2.5	12.18	(8.35, 15.54)	.002
Age	−1.45	.24	(.11, .34)	< .001
Weight	−1.38	.25	(.11, .43)	.002
Sex of a child (Ref. = Male)				
Female	−1.86	.16	(.13, .45)	.005
Residence area (Ref. = Rural)				
Urban	−1.85	.16	(.12, .43)	.025
Gestational age(Ref. = late preterm)				
Extermly preterm	.03	1.35	(1.06, 1.67)	0.01
Very preterm	.05	1.05	(1.02, 1.45)	.002
Working status of a child (Ref. = yes)				
No	.85	2.34	(1.12, 4.45)	.021
Breast feeding history(Ref. = Yes)				
No	.35	1.419	(1.13, 1.67)	<.000
Vaccination history(Ref. = Yes)				
No	.28	1.323	(1.18, 1.56)	.004
Additional disease history(Ref. = Yes)				
No	−0.75	.47	(.13, .61)	.002
Mother’s nutritional status(Ref. = under-weight)				
Normal	−.54	.58	(.13, .85)	.003
Parent’s level of education(Ref. = Secondary and above)				
No education	.65	1.92	(1.52, 4.58)	.012
Primary	.23	1.26	(1.08, 3.46)	.023
Mother’s stature(Ref. = Normal)				
Short	.74	2.10	(1.13, 4.45)	.024
Mother’s occupation (Ref. = Household wife)				
Gov’t employee	.65	1.92	(1.32, 2.35)	<.001
Private worker	.07	1.07	(1.01, 1.38)	.01
Mother’s marital status(Ref. = married)				
Others	.87	2.39	(1.13, 3 .46)	.032
HH monthly income (Ref. = less than 500)				
500–1000	−.09	.92	(.43, .99)	.024
> 1000	−.93	.39	(0.12, 0.57)	.01
Age range between children(Ref. = < 2 years)				
> 2 years	−2.78	.062	(.132, .452)	< 0.061
Family size in HH(Ref. = greater than 10)				
< 5			(0.23, 0.87)	.003
6–10	−.45	.64		
	−.02	.98	(.45, .99)	.021
No of under-five children in HH(Ref.= > 2)				
> 2	−.32	.72	(.35, 0.92)	.002
Source of adquate HH food(Ref. = yes)				
No	.02	1.02	(1.01, 1.32)	.013
Source of improved drinking water (Ref. = yes)				
No	.04	1.04	(1.01, 1.32)	.023
Moderate cooking fuel(Ref. = yes)				
No	.02	1.02	(1.001, 1.081)	.021
Ownership of Livestock(Ref. =yes)				
No	.43	1.53	(1.12, 1.92)	.001
Appetite test(Ref. = Pass)				
Fail	.12	1.13	(1.02, 1.45)	0.021
Age*Sex(Ref. = Male)				
Female	.05	1.051	(1.089, 1.435)	<.001
Age *Residence area(Ref. = Rural)				
Urban	.28	1.323	(1.221,1 .445)	.002
Weight *mother;s breast milk feeding(Ref. = Yes)				
No	.46	1.584	(1.038, 1.725)	0.003

As it is indicated in Table 4, the average recovery time from SAM for female children was decreased by 75% (HR = .25, 95% CI: (.11, .43), p-value = 0.002) as compared to male children and the average recovery time from SAM for urban children was decreased by 84% (HR = .16, 95% CI: (.13, .45), *p*-value = .025) as compared to children with vomiting. Similarly, the average recovery time from SAM for children with extremely preterm gestational age was increased by 35% (HR = 1.35, 95% CI: (1.06, 1.67), *p*-value = 0.01) as compared to late preterm children, keeping the other conditions constant. The average recovery time of working status children was decreased by 84% (HR = .16, 95% CI (.12, .15), *p*-value = 0.021). The average recovery time of a child with normal mother’s nutritional status was decreased by 42% (HR = .52, 95% CI (.13, .85), *p*-value = .003). As age of a child increased by one month, the average recovery time of a child from SAM was decreased by 76%(HR = .24, 95% CI; (.11, .34), *p*-value< .01) given the other covariates constant. Similarly, as weight of a child increased by one kg, the average recory time of a child from SAM was decreased by 75% (HR = .25, 95% CI;(.11, .45), *p*-value = .002).

Parents level of education had also significant effect for average recovery time of children from SAM. Hence, the average recovery time a child with non educated parent was increased by 92% (HR = 1.92, 95% CI; (1.52, 4.58), *p*-value = 0.12) as compared to educated parents, keeping the other covariates constant. The average recovery time of a child whose house hold income greater than 1000 per month was decreased by 61% (HR = .39, 95% CI; (0.12, 0.57), *p*-value = .01) as comapred to average recovery time of a child whose house hold income less 500 per moth. The average recovery time from SAM for a child who did not get adquate HH food was longer by 2%(HR = 1.02, 95% CI;(1.01, 1.32), *p*-value = 0.013) as comapred to a child who got adquate HH food, keping the other things constant. The average recovery time from SAM for a child whose family had less than 2 under five chilred was shorter by 28%(HR = .72, 95% CI; (.35, 0.92), *p*-value = .002).

The average recovery time from SAM for a child who did not get improved drinking water was longer by 4%(HR = 1.04, 95% CI;(1.01, 1.32), *p*-value = 0.023) as comapred to a child who got improved drinking water, keping the other things constant. Similarly, the average recovery time from SAM for a child who did not get moderate cooking fuel was longer by 2% (HR = 1.02, 95% CI; ((1.001, 1.081), *p*-value = .021) as compared to those children coame from families with access of moderate cooking fuel, keeping the other covariates constant.

The average recovery time from SAM for children whose mothers are government employee was longer by 92% (HR = 1.95, 95% CI; (1.32, 2.35), *p*-value < 0.01) as compared to those children whose mothers occupation is house hold wife.

Breast feeding and vaccination histories also significantly associated with differences of recovery time of children from SAM. Hence, the average recovery time from SAM for a child who did not get his/her mothers’ milk at least for the first six months after born, was increased by 41.9%(HR = 1.419, 95% CI: (1.13, 1.67), *p*-value <.001) as compared to children who got mothers milk, keeping the other variables constant. The average recovery time from SAM for children who did not get immunization during their child hood was increased 32.3% (HR = 1.323, 95% CI: (1.18, 1.56), *p*-value = .004) as compared to children who got immunization at their child hood. With similar interpretations, the variables like age between children, source of adequate HH food, size of families and under-five children per families had significant effect on the variable of interest (Refer to Table 4),

In addition to the main effects, important interaction effects were statistically significant for average recovery time of children from SAM. In Table 4, three interaction effects namely age * sex, age*residence area and weight *breast feeding history were statistically significant for the variable of interest (average recovery time).

### Interaction effect of age and sex of children

As it is indicated in Table 4, as age of children increased by one month, the average recovery time of children was decreased by 76.5% (HR = .235, 95% CI:(.109, .342), p-value<.001) keeping the other factors constant. However, the decreasing rate of males and females were not the same, hence, as age increased by one month, the decreasing rate of female children was increased by 5.1% as compared to male children (HR = 1.051, 95% CI = (1.089, 1.435), *p*-value < 0.001).

#### Interaction effect between age and residence area

As it is indicated in Table 4, as age increased by one month, the decreasing rate of urban children was increased by 32.3% as compared to rural children (HR = 1.323, 95% CI = (1.221, 1 .445), *p*-value = 0.002).

#### Interaction effect between weight and breast-milk feeding history

As it is indicated in Table 4, as weight increased by one kg, the average recovery time of children decreased by 74.8% (HR = .252, 95% CI:(.112, .425), *p*-value = .002) keeping the other factors constant. However, the decreasing rate of children who fed exclussive breast milk at childhood and those who didn’t get exclussive breast milk were not the same, hence, as weight increased by one kg, the decreasing rate of children who fed mothers’ exclussive breast milk was increased by 58.4% as compared to those children who did not fed mother’s breast milk (HR = 1.584, 95% CI = (1.038, 1.725), *p*-value = 0.003).

## Discussion

In current investigation, the average recovery time of children from SAM was estimated. The statistical significant variables for difference of recovery time of different groups were also investigated. The assessment was conducted for association between the variable of interest (average recovery time) and predictors. Hence, the average recovery time of children from SAM was found to be 21 days. Eventhough, the value is with in the international standards set < 28 days, it is long period as compared to other studies in different areas [10].

The finding is contradicted with other retrospective previous studies conducted at Bahir Dar, North west Ethiopia with result 18 days and a research conducted in Shebedido woreda (southern Ethiopia) which declared that the average recovery time was 19 days [11, 16]. However, a research conducted in Zambia indicates that the average recovery time from SAM was 13 which is significantly less than a result obtained in current investigation. The potential reason for this difference might be differences in treatment practice, health care surroundings, socio-demographic, economic and related factors in the study areas [11].

Difference in recovery time of children from SAM with additional diseases obtained in this study is consistent with other previous studies. Hence, children with SAM having additional disease needs more recovery time as compared to those children with no disease [14].

Children admitted in the treatment site because of SAM who come from low income families, families living in traditional house, families who are not using moderate cooking fuel, families who do not have any access of improved drinking water, children born from non-educated parents and children born from his mother with less than 2 years interval do not recover from SAM with short period of time. Such children need some extra time to be recovered from the disease. This finding agrees with a result obtained in previous research [21].

The number of families, especially, number of under-five children per families affect the average recovery time from SAM. Hence, the larger size in number of under-five children, the longer the time it needs for a child to be recovered from SAM. The potential reason for this might be no more attention/care is given for a child because of another under-five children. More family size particularly, unemployed families lead for shortage of adequate HH food. This finding is also consistent with a result obtained from previous investigations [22, 23].

The result obtained in this research indicates that, children with SAM who had additional disease requires more time to be recovered from SAM as compared to those children who are free from additional disease. This result is contradicted with a research conducted in Burkina Faso [24] which states that additional disease (Anemia) has no negative impact on recovery time and the research agrees with another study conducted in Ethiopia [16]. The potential reason for this difference might be health care surroundings and other socio-economic determinants among children in the study areas.

### Conclusion

The average recovery time of children from SAM in the study area was 21 days, which is really long period of time compared with researches conducted in any other areas and different groups with different characteristics. Weight of a child at birth, gestational age of a child, working status of a child at admission, birth order of a child, mother’s BMI, parent’s level of education, mother’s stature, mother’s occupation, mother’s age, mother’s marital status, mother’s nutritional status, house hold income in ETB, family size in HH, number of under-five children, the type of toilet used in HH, the type of house families live, source of improved drinking water, type of cooking fuel, sex of a child, residence area, malnutrition type, age of a child at admission and weight of a child were identified as predictors for the variation of average recovery time from SAM.

As recommendation**, t**he area, where current investigation was conducted needs special intervention for children to be free from stunting, wasting and under-weighting. The children faced with lack of balanced diet and this further leads to be affected by the different diseases.

Awareness should be created to the community to feed mothers’ breast milk, to use moderate cooking fuel, to use improved drinking water, to have few numbers of children per family (family planning) and vaccinate their children at child hood and vaccination program should be continued with large coverage including rural areas. Attention should be given for children with additional disease and a child who come from families who have more children with malnutrition status. More attention should be given for those children who did not start to reduce their edema during their follow up time and for those children who can’t pass to phase2 during their follow up time.

This study has both theoretical and methodological contributions. The interaction effect between covariates can be considered as theoretical contribution. According to the analysis given in current study, suggestions are given to improve the average recovery time of children from SAM in study area and enhance the equality in variable of interests between different groups (male and female, urban and rural, children with families of different level of education, children with families of different economic and living standared). Hence, from a policy point of view, the main findings of current study suggest that a special attention should be given for children who have londer waiting time to be recovered from SAM which is an indication of practical contribution.

## Data Availability

The data used for current investigation is availiable under corresponding author.

## Abbreviations

SAM: Severe Acute Malnutrition
HIV: Human Immune deficiency virus
AHR: Adjusted Hazarded Ratio
CI: Confidence interval
RUTF: Ready to use therapeutic food
MUAC: Mid-upper-arm circumstance
SPSS: statistical packages for social science.
